# Effectiveness of Telerehabilitation Interventions for Self-management of Tinnitus: Systematic Review

**DOI:** 10.2196/39076

**Published:** 2023-02-09

**Authors:** Sara Demoen, Antonios Chalimourdas, Annick Timmermans, Vincent Van Rompaey, Olivier M Vanderveken, Laure Jacquemin, Winfried Schlee, Wim Marneffe, Janis Luyten, Annick Gilles, Sarah Michiels

**Affiliations:** 1 Rehabilitation Research Center Faculty of Rehabilitation Sciences Hasselt University Hasselt Belgium; 2 Department of Otorhinolaryngology and Head and Neck Surgery Antwerp University Hospital Antwerp Belgium; 3 Department of translational neurosciences Faculty of Medicine and Health Sciences University of Antwerp Antwerp Belgium; 4 Department of Psychiatry and Psychotherapy University of Regensburg Regensburg Germany; 5 Faculty of Business Economics Hasselt University Hasselt Belgium; 6 Department of Education, Health and Social Work University College Ghent Ghent Belgium

**Keywords:** tinnitus, audiology, systematic review, telerehabilitation, self-management, intervention, quality of life, internet, self-help, mobile phone

## Abstract

**Background:**

Tinnitus is a highly prevalent symptom affecting 10%-20% of the adult population. Most patients with tinnitus have chronic tinnitus, which can directly or indirectly disrupt their daily life and negatively affect the health-related quality of life. Therefore, patients with tinnitus are frequently in need of costly and time-consuming treatments. As an answer, telerehabilitation interventions are on a rise to promote self-management in patients with tinnitus and reduce their dependency on in-person care.

**Objective:**

This systematic review aimed to provide an overview of the research concerning the effectiveness of telerehabilitation interventions for self-management of tinnitus.

**Methods:**

This systematic review adheres to the PRISMA (Preferred Reporting Items for Systematic Reviews and Meta-Analyses) guidelines. Studies were eligible for inclusion if study participants were adult patients with complaints of primary subjective tinnitus and the study intervention comprised any possible telerehabilitation form for the self-management of tinnitus complaints. A search for eligible studies was conducted on PubMed, ScienceDirect, Scopus, Web of Science, and Cochrane Library. The Cochrane Risk of Bias 2 tool was used to the assess risk of bias.

**Results:**

In total, 29 articles were found eligible, and of these, 5 (17%) studied multiple telerehabilitation forms. Internet-based cognitive behavioral treatment with guidance by a psychologist or audiologist was examined in 17 studies (n=1767), internet-based cognitive behavioral treatment without guidance was examined in 4 studies (n=940), self-help manuals were examined in 1 study (n=72), technological self-help devices were examined in 2 studies (n=82), smartphone apps were examined in 8 studies (n=284), and other internet-based interventions were examined in 2 studies (n=130). These rehabilitation categories were proven to be effective in decreasing tinnitus severity and relieving tinnitus distress as measured by tinnitus questionnaires such as Tinnitus Functional Index, Tinnitus Handicap Inventory, or Tinnitus Reactions Questionnaire. However, dropout rates were often high (range 4%-71.4%). All studies reported between some concerns and high concerns of risk of bias, resulting in low to moderate certainty levels.

**Conclusions:**

Overall, there is low to moderate quality evidence that telerehabilitation interventions effectively reduce tinnitus severity and distress. These interventions form a possible tool to improve the self-management capacities of the patient and the accessibility of tinnitus care as a replacement or an addition to in-person care. Nevertheless, barriers such as lack of time, engagement, motivation, and openness of the patient causing high dropout should be considered.

**Trial Registration:**

PROSPERO International Prospective Register of Systematic Reviews CRD42021285450; https://www.crd.york.ac.uk/prospero/display_record.php?RecordID=285450

## Introduction

### Background

Tinnitus is often referred to as a buzzing, ringing, or hissing sound perceived in one or both ears or centrally within the head. It can be defined as the perception of a constant or intermittent sound without the presence of external auditory stimuli [1-3]. Tinnitus is a very common symptom, with a prevalence of 10%-20% of the world population, which accounts for >70 million people solely in Europe [1,2,4,5]. Most patients with tinnitus have chronic subjective tinnitus, that is, they have experienced tinnitus for ≥6 months, and the tinnitus sound is a phantom sensation of sound without the presence of a physical sound source and is hypothesized to be owing to abnormal neural activity [6-8]. The underlying cause of tinnitus varies widely and can be of an auditory as well as a nonauditory nature [9,10]. Consequently, there is also great variance in the clinical presentation. Some patients can easily ignore the tinnitus sound and do not find it bothersome, while 5%-10% of the patients with tinnitus experience substantial disruptions of daily functioning and health-related quality of life [4,11,12]. These differences are also because of the diversity in comorbidities and associated complaints such as mood changes, anxiety, depression, sleep disorders, concentration problems, and other psychological or emotional issues [4,11,12].

There is no standard rehabilitation trajectory for the treatment of tinnitus as no single tinnitus treatment is beneficial for every patient [13-16]. *The tinnitus profile* of each patient is unique and needs a tailored, multidisciplinary treatment approach, which can consist of a combination of several treatment forms [13-16]. The current clinical management strategies that are most often recommended include education, counseling, tinnitus retraining therapy (TRT), or cognitive behavioral therapy (CBT) [16,17]. TRT is a combination of sound therapy and retraining counseling, teaching patients about the auditory system and the mechanisms by which tinnitus is thought to arise [18-21]. It aims to induce habituation of tinnitus-induced reactions and tinnitus perception, allowing patients to achieve control of their tinnitus [22]. CBT can consist of multiple aspects such as applied relaxation, positive imagery, cognitive restructuring of negative beliefs about tinnitus, exposure to sounds, behavioral activation, and mindfulness and attention exercises [6,21,23,24]. CBT is often guided by a psychologist who offers support and provides tools to deal with tinnitus [6,21,23,24]. Both TRT [18-21] and CBT [6,21-23] are proven to be effective for alleviating the complaints of tinnitus. However, while both these treatments are effective, they are also costly and time consuming, especially if patients are unable to maintain results and are repeatedly in need of help in whichever form [25-27]. Treatments reducing the need for in-person care by increasing patients’ self-management skills are therefore of high interest.

To address the burden of in-person care of patients with tinnitus, self-management, self-help, and low-contact treatment forms are on the rise. Partially owing to the COVID-19 pandemic, telerehabilitation, that is, delivery of care from a distance, became a necessity rather than an opportunity [28-30]. Studies have shown that low-contact treatment provided from a distance through applications and videoconferencing can be used as a substitution of or an addition to in-person clinical care for several conditions [31-36]. Although real-time telerehabilitation with synchronous video consultations is the most commonly known and used form of telerehabilitation [35,37], this field is not restricted to that. Telerehabilitation is remotely delivering care with any form of technology; this can indeed be by using audio or video communication but also by making use of messaging platforms for guidance, software in smartphone apps with rehabilitative purposes, virtual reality, or a combination of all these forms of technology [38,39]. In the field of audiology and, more specifically, in the field of tinnitus, telerehabilitation in both full and hybrid forms has the potential to be very useful during the entire therapeutic process [29,40,41]. Telerehabilitation has been used as an aid for early screening, initial evaluation, diagnosis, therapy, long-term monitoring, provision of web-based support, etc [40]. For example, in the last decade, computer platforms and smartphone apps increasingly found their way into the treatment of tinnitus, most often in the form of CBT and sound therapy [42,43]. These apps are easy to implement in clinical practice and form easily accessible treatment options. In addition, they might also improve the cost-effectiveness of tinnitus treatment [40,43,44]. As telehealth is a highly dynamic research field, the treatment options for tinnitus in the future world of telerehabilitation will progress even more owing to the advances made in the field of technology [44].

### Objectives

In the past decades, various low-contact and self-management telerehabilitation treatments were developed for patients with tinnitus to search for an alternative or additional tool for the existing treatment options that are time consuming and costly. The current review aims to make an overview of the research concerning the effectiveness of telerehabilitation interventions for self-management of tinnitus.

## Methods

This systematic review adheres to the PRISMA (Preferred Reporting Items for Systematic Reviews and Meta-Analyses) guidelines [45]. The predefined protocol was registered in the International Prospective Register of Systematic Reviews (PROSPERO; CRD 42021285450).

### Eligibility Criteria

The eligibility of the studies was assessed based on the inclusion and exclusion criteria listed in Textbox 1. Studies were included if participants were adult patients (aged >18 years) reporting subjective tinnitus as a primary complaint. The distinction whether the subjective tinnitus of the study participants was either primary or secondary was made after screening the full text as this information is often not included in the abstracts of the studies. The study intervention had to include any possible telerehabilitation intervention for the self-management of tinnitus complaints. Synchronous telerehabilitation interventions, where the remote face-to-face intervention mimics in-person care, were not considered for this review as these interventions do not aim to improve the self-management of patients. The search concluded on October 18, 2021, and no publication date restrictions were imposed.

**Eligibility criteria.**

- Inclusion criteria
  Adult patients (aged >18 years)
  Subjective tinnitus as a primary complaint
  A study intervention comprising any possible form of self-management or telerehabilitation
  No publication date restrictions
- Exclusion criteria
  No tinnitus
  Objective tinnitus
  Tinnitus as a secondary complaint
  Full text not available in Dutch, French, German, Greek, or English

### Information Sources

We consulted PubMed (National Center for Biotechnology Information), ScienceDirect, Scopus, Web of Science, and Cochrane Library in the search of eligible studies.

### Search Strategy

The search queries were composed using the terms “tinnitus (population) and self-management,” “telerehabilitation,” “smartphone app (intervention),” and their synonyms. Both free terms and Medical Subject Headings terms were included in the search. The complete search queries are included in Multimedia Appendix 1.

### Selection Process

**Figure 1:**
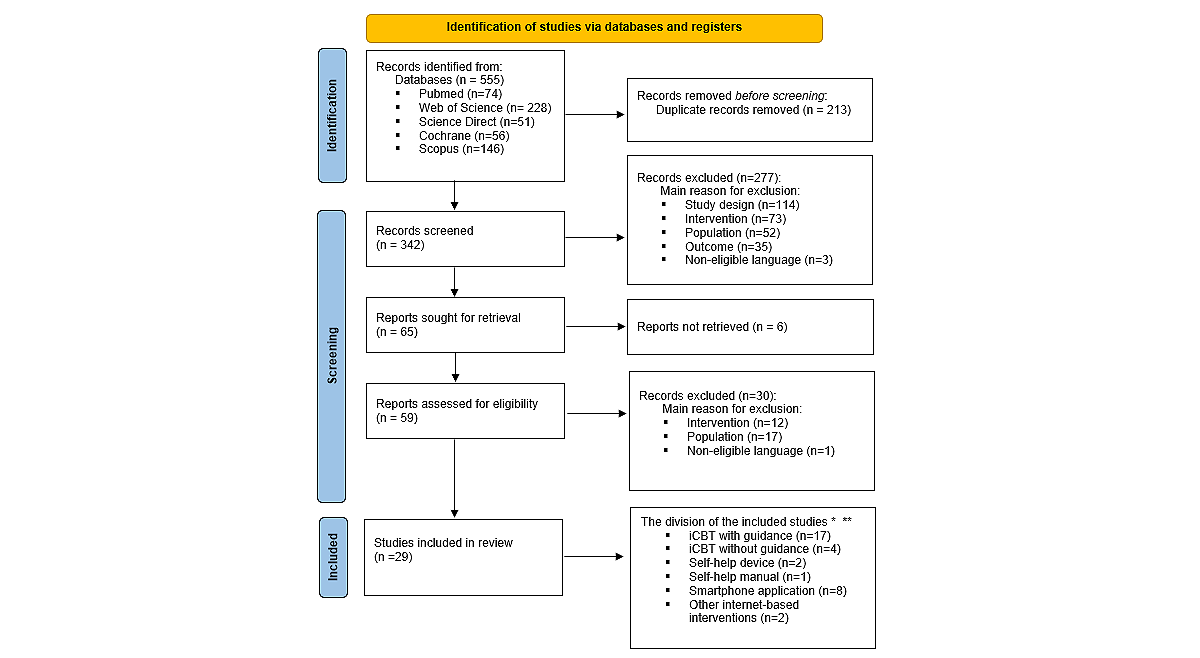
Flow diagram for study inclusion (PRISMA [Preferred Reporting Items for Systematic Reviews and Meta-Analyses] 2020 [47]). iCBT: internet-based cognitive behavioral therapy. *some overlap between studies was possible, since some studies examined more than one form of telerehabilitation. **subgroups of included studies based upon form of telerehabilitation.

All articles collected by the search strategy were screened twice. Two reviewers (SD and AC) independently and blindly performed the eligibility assessment on the title and abstract [46]. Afterward, the blind mode was turned off, and disagreements were discussed and resolved during a consensus meeting (SD, AC, and SM). Articles that were included by both reviewers or received a label “maybe” were considered for the second screening for full text. This second screening was also fulfilled independently by the 2 researchers. After a second consensus meeting and cross-referencing the reference lists of the included articles (SD, AC, and SM), the final articles were obtained (Figure 1) [45].

### Data Collection

The included articles were divided into subcategories based upon the different forms of telerehabilitation. In total, 6 subcategories were distinguished, including internet-based CBT (iCBT) with guidance (feedback, monitoring, and support by a psychologist or audiologist), iCBT without guidance, self-help devices, self-help manuals, smartphone apps, and other internet-based interventions. For all studies, information was collected and arranged in data extraction tables per subcategory. Information was organized according to the applicable items of the subdivisions: population, intervention, comparison, and outcome.

### Data Items

The recorded study and population characteristics included author, year of publication, study design, sample size (n), gender distribution (%), mean age and SD (years), type of tinnitus, mean duration of tinnitus, and presence of hearing loss.

The tables of evidence per subcategory had 3 main categories: intervention and comparison, outcome, and results. These categories are subdivided as follows:

1. Intervention (+ if applicable comparison: control intervention): the used intervention form and specifics, such as guidance, duration, and follow-up, are indicated.
2. Outcome: outcome measures and dropout are the items discussed.
3. Results: data results, Grading of Recommendations Assessment, Development, and Evaluation (GRADE) score, and the conclusions are listed.

The tables were subdivided per subcategory of self-management and telerehabilitation and, in addition, per study design, that is, within each subcategory the studies are separated by trial status.

### Risk of Bias Assessment

For the risk of bias (RoB) assessment, the Cochrane RoB2 tool was used. This tool is specifically designed for assessing RoB in randomized controlled trials (RCTs) [47]. However, the domains are, in general, also applicable for other study designs [47]. Most of the included studies are RCTs, and the remaining studies are of diverse study design, making it difficult to compare RoB. Therefore, it was decided to use the RoB2 tool for all included studies [47]. The figures summarizing the RoB conclusions, per domain and overall, were made using RoBvis, an RoB visualization tool by Cochrane [48].

### Data Synthesis

Owing to the heterogeneity in telerehabilitation forms and the differences in chosen outcomes and methodology of the included studies in this review, the reviewers opted not to perform a meta-analysis but to summarize the data in a systematic review. Data were discussed according to the subdivisions mentioned under the *Data Items* section.

### Certainty Assessment

Certainty of study results was examined using the GRADE framework considering the RoB, imprecision, inconsistency, indirectness, and publication bias of the included studies [49].

## Results

### Study Selection

A total of 555 potentially relevant articles were retrieved from the initial database searches on October 18, 2021 (Figure 1 [45]). Of these 555 articles, 213 (38%) duplicates were removed, resulting in 342 (62%) remaining articles for primary screening. In the primary screening, 81% (277/342) of articles were excluded. The reasons for exclusion are specified in Figure 1. Finally, the full text screening was completed for 59 articles as the full text of remaining 6 articles could not be retrieved. Of these 59 articles, 30 (51%) articles were excluded as 12 (40%) articles were based upon wrong intervention (no telerehabilitation), 17 (57%) articles were based on wrong population (no primary tinnitus complaint or patients aged <18 years), and 1 (3%) article was excluded because of the full text being in a noneligible language. This resulted in a total of 29 eligible articles [50-78]. These articles were divided into subgroups based upon the used form of telerehabilitation as an intervention. Overall, 6 studies were categorized into 2 subgroups, as they compared 2 intervention forms. The level of agreement between both reviewers during the study selection process was 93% for the primary screening and 96% for the screening on full text.

### RoB Assessment

**Figure 2:**
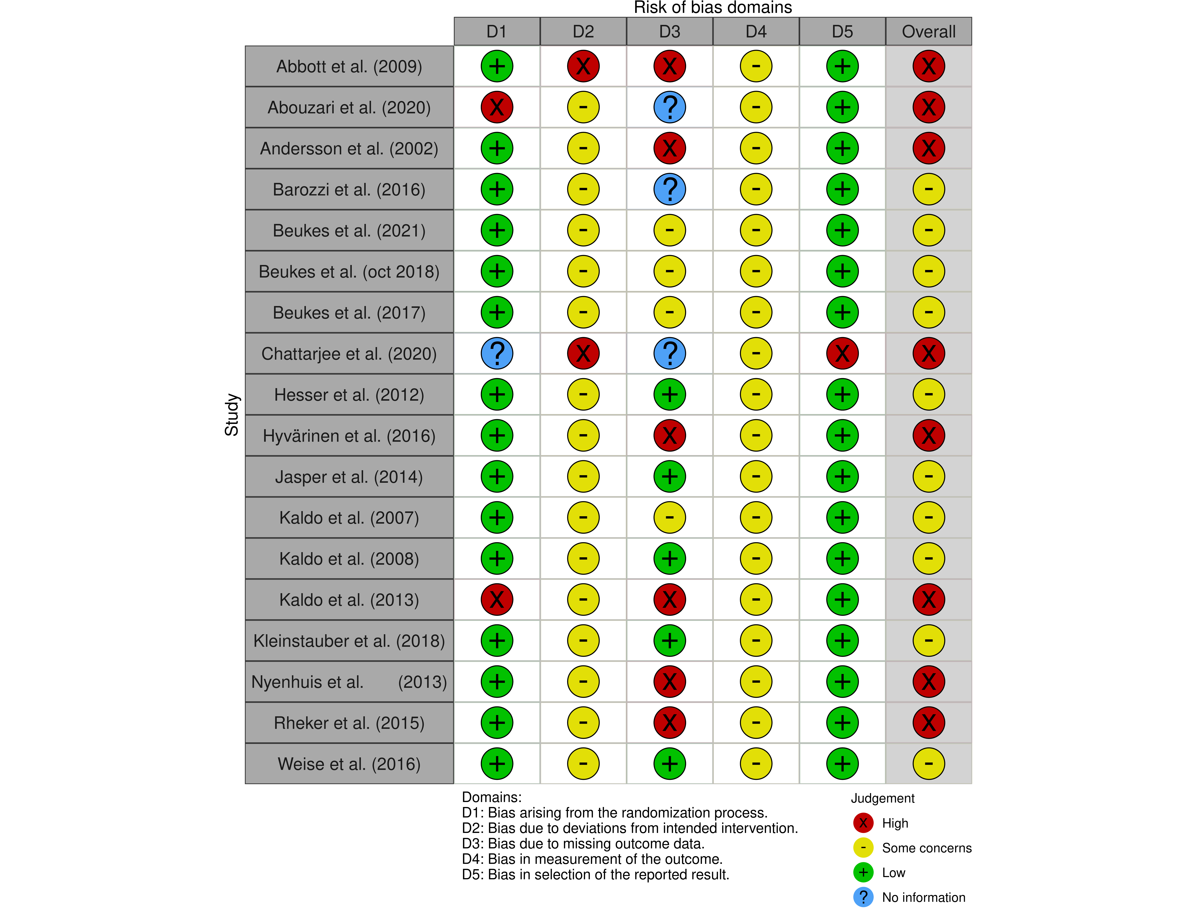
Risk of bias controlled trials scoring per domain.

Most of the controlled trials were RCTs with a low RoB arising from the randomization process (D1) [50,53,55,56,58-60, 63,64,66-70,73,74]. Two studies were non-RCTs [61,72]. All controlled trials showed some reasons for concern of RoB owing to deviations from the intended interventions (D2), except for Abbott et al [50] and Chattarjee et al [74]. RoB owing to missing outcome data (D3) was scored “high concerns” and “some concerns” for studies with respectively moderate to high dropout rates and no clearly specified plan on how to handle these missing data. The 3 controlled trials concerning smartphone apps did not provide any information concerning missing data and dropout [72-74]. All controlled studies were rated “some concerns for RoB” in the measurement of outcome (D4) as assessors were not blinded. Most studies did have a full, clear, correct, and specified plan for statistical analysis, suggesting that the concern for RoB in the selection of the reported result (D5) was low. Overall, studies were rated “some concerns” to “high concerns” of RoB. Figure 2 provides the RoB assessment results of the controlled trials.

**Figure 3:**
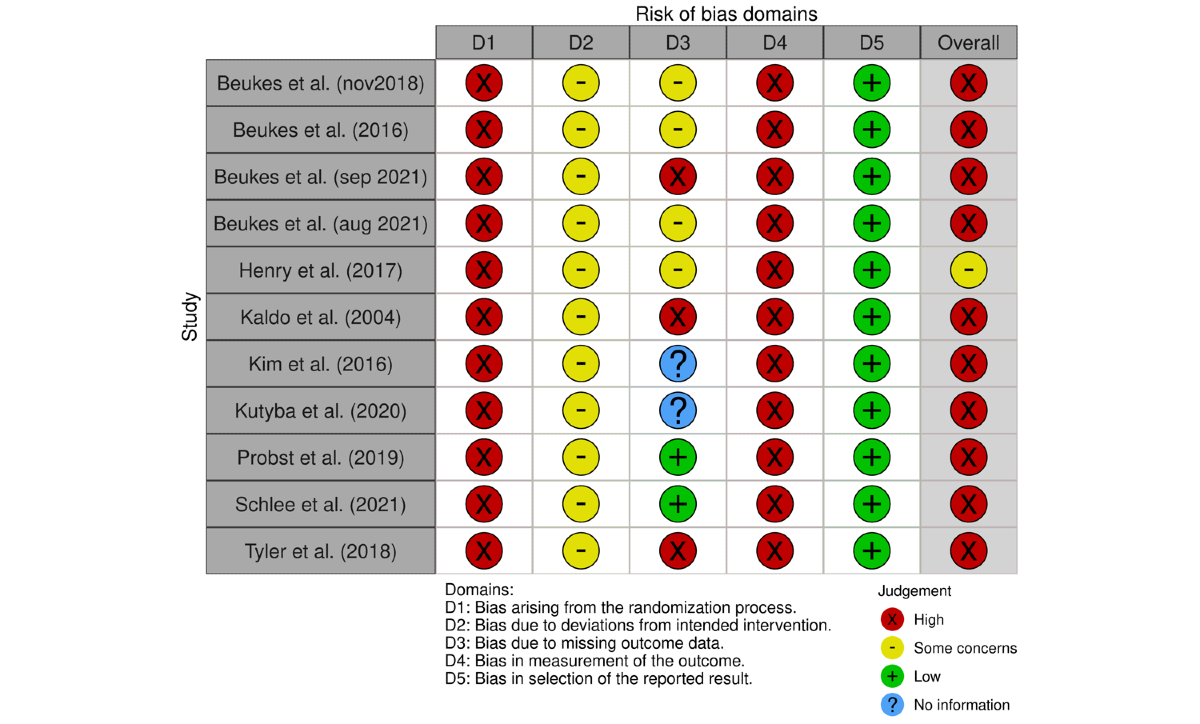
Risk of bias noncontrolled trials per domain.

They did all show some concerns of RoB owing to deviations from the intended interventions (D2). RoB because of missing outcome data (D3) was again scored “high concerns” and “some concerns” for studies with respectively moderate to high dropout rates. All studies specified their statistical analysis beforehand, resulting in low RoB in the selection of the reported result (D5). Figure 3 provides the RoB assessment results of the noncontrolled trials.

A summarization of the RoB scores can be found in Figures S1 and S2 in Multimedia Appendix 2.

### Certainty

In general, the included studies had a low 

 to moderate 
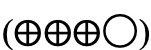
 certainty level according to the GRADE framework [49]. GRADE scores are indicated for each study.

### iCBT With Guidance

#### Characteristics of Included Studies

Most of the eligible articles, more specifically 17 studies [50-67], dealt with iCBT with guidance in a population of adult patients with chronic tinnitus. The guidance offered to the patients was often feedback, monitoring and support through telephone conversation, email, or a web-based messaging system within the platform. Most often, the guidance was provided by a psychologist, except for the 7 studies by the research group of Beukes [51-57]. They chose audiologists trained in iCBT to provide the required guidance. The studies were published within the time period of 2004 to 2021 and comprised a total study sample of 1767 participants, of which 929 participants received iCBT with guidance. Table S1 in Multimedia Appendix 3 [50-78] provides further details of the included studies.

#### Results of Individual Studies

All iCBT with guidance programs were based upon the principles explained in the self-help manual by Andersson and Kaldo [79]. However, not all iCBT programs were structured in the same manner. The iCBT programs within the studies varied in terms of how the content was divided into components or modules, adaptations in the content of the iCBT, and the duration of the program. The studies indicated to make the iCBT programs interactive by using homework assignments, quizzes, diaries, or worksheets. Patients were also given the possibility to dive deeper into some of the CBT principles for tinnitus by additional optional modules.

The primary outcome of all studies was tinnitus severity or distress, which was measured by the Tinnitus Functional Index (TFI), Tinnitus Reaction Questionnaire, or Tinnitus Handicap Inventory (THI). Various secondary outcome measures, using self-report questionnaires, were appended. Tables 1 and 2 provide further details of the included studies. Most of the studies concluded that the iCBT, with guidance provided by either a psychologist or an audiologist trained in iCBT principles, was effective in the treatment of chronic tinnitus. However, Abbott et al [50] and Beukes et al [55] concluded that iCBT was not effective or that a valuable conclusion could not be drawn owing to extremely high dropout rates (71.4% and 52.4%-69.8%, respectively) in the intervention groups receiving iCBT. An important note to be made is that other studies that reported iCBT to be effective in reducing tinnitus distress also showed dropout rates ranging from 4% up to 37% after treatment and from 6% up to 50% at follow-up.

**Table 1:** Table of evidence: internet-based cognitive behavioral therapy (iCBT) with guidance (controlled trials).

Author, year	Intervention	Guidance	Comparison	Duration intervention	FU^a^	Outcome measures	Dropout	Data results	Conclusion+ GRADE^b^ score
Abbott et al [50], 2009	iCBT: 10 components in 6 modules + homework assignments + weekly diaries	Weekly feedback through mail	TIP^c^ psychoeducation + weekly MCQs^d^	6 weeks	N/A^e^	SITHC^f^ (9 questions + CITD^g^), TRQ^h^, DASS^i^, WHOQOL-BREF^j^, OSI-R^k^, VAS^l^ tinnitus loudness, annoyance, control over tinnitus, and QOS^m^, TCS^n^-modified, TSQ^o^-modified	IG^p^: 71.4%; CG^q^: 30.4%	No significant main effect or interactions were found (*P*>.05), except a significant time effect for PSQ^r^ (*P*=.03)	iCBT not effective and suitable in Australian industrial population  ^s^
Beukes et al [55], 2021	iCBT: 22 modules with explanatory videos + homework assignments + worksheets + quizzes	Guidance by audiologist: outlining content, monitoring progress, providing feedback, questions + encrypted 2-way message system within ePlatform	iCBT: applied relaxation 10 modules of CBT and after 4 weeks the remaining 12 modules of iCBT	T1: after 4 weeks	T2: after 8 weeks; T3: 2 months FU	TFI^t^ (primary), GAD-7^u^, PHQ-9^v^, ISI^w^, TCQ^x^, EQ-5D-5L^y^, THS^z;^ compliance	T1/T2—IG: 52.4%, CG: 55.6%; T3—IG: 69.8%, CG: 63.5% did not complete assessment	59% of IG and 56% of CG experienced a significant change of 13 points on the TFI at T1, 65% of IG and 62% of CG at T3	Owing to low compliance, no generalizable conclusion could be drawn ⨁⨁◯◯
Beukes et al [53], 2018	iCBT: 16 recommended modules + 5 optional modules + homework assignments + worksheets+ quizzes	Guidance by audiologist: outlining content, monitoring progress, providing feedback, questions (minimal 10 minutes/week) + encrypted 2-way message system within ePlatform	Face-to-face standard information counseling generally used in the United Kingdom as tinnitus treatment	8 weeks (2 modules/week + 1 extra optional module each week between week 2 and 6)	2 months	TFI (primary), THI-S^aa^, GAD-7, PHQ-9, ISI, HHIA-S^ab^, HQ^ac^, CFQ^ad^, SWLS^ae^	T1—IG: 4.3%, CG: 4.3% T2—IG: 19.6%, CG: 19.6% did not complete assessment	IG had greater weekly reductions in tinnitus distress THI-S and TFI	iCBT and standard face-to-face information counseling are equally effective for reducing tinnitus distress and most tinnitus-related difficulties 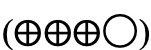 ^a^^f^
Beukes et al [56], 2017	iCBT: 16 recommended modules + 5 optional modules + homework assignments + worksheets + quizzes	Guidance by audiologist: outlining content, monitoring progress, providing feedback, answering questions, (minimal 10 minutes/week) + encrypted 2-way message system ePlatform	8 weeks wait-list	8 weeks (2 modules/week + 1 extra optional module each week between week 2 and 6)	2 months	TFI (primary) THI-S, GAD-7, PHQ-9, ISI, HHIA-S, HQ, CFQ, SWLS	T1—IG: 13.7%, CG: 1.4%; T2—IG: 26%, CG: 17.8% did not complete assessment	51% of IG experience a significant change of 23.3 points on the TFI and showed a statistically significant greater reduction in TFI than CG (Cohen *d*=0.7; *P*<.001)	Guided iCBT for tinnitus using audiological support resulted in statistically significant reductions in tinnitus distress TFI 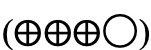
Hesser et al [58], 2012	IG1: iCBT: 8 modules+ exercises and homework assignments; IG2: iACT^ag^	Feedback provided by psychologist on homework assignments through contact handling system + encouragement	CG: discussion forum specifically targeting tinnitus-related problems	8 weeks	1 year	THI (primary), HADS^ah^, ISI, QoLI^ai^, PSS^aj^, TAQ^ak^	In total: 4% after treatment and 6% at FU	Significant effects where noted on THI for both iCBT and iACT compared with control after treatment	iCBT and iACT are both equally effective 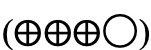
Jasper et al [59], 2014	iCBT: 12 mandatory and 6 optional text modules + suggestion exercising + work sheets + solutions for common problems	Communication through secured encrypted webpage with psychologist once a week (+ max 10-minute email/week)	CG1: group CBT: 10 weekly sessions of 90 minutes in groups of 5-12 patients by psychologist CG2: discussion forum	10 weeks	6 months	THI (primary outcome), mini-TQ^al^, HADS, ISI, TAQ; Credibility and expectancy questionnaire	After treatment: IG1: 7.3%, IG2: 7.0%, CG: 2.3% at 6 months FU: IG1: 17.1%, IG2: 14%	Patients receiving iCBT (IG) or gCBT^am^ (CG1) showed significantly reduced tinnitus distress compared with participants of DF (CG2)	iCBT might be equally effective as gCBT in the management of chronic tinnitus 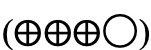
Kaldo et al [63], 2008	iCBT: 6-step treatment program, each step consisting of 1-4 modules + 16 mandatory tools + homework + worksheets + diary	Guidance by psychologists through email to give feedback, support, give recommendations, foster adherence, or help out with problems	Group CBT: 7 weekly sessions of 2 hours in groups of 6-7 patients by psychologist + same self-help material + worksheets	6 weeks for iCBT and 7 weeks for gCBT	1 year	TRQ (primary outcome), THI, VAS tinnitus loudness, VAS tinnitus distress, VAS perceived stress, HADS, ISI	In total not specified per group: 4% after treatment and 13% at FU	38% of IG had a clinically significant change in tinnitus distress after treatment and 35% at FU, for CG this was 44% after treatment and at FU	Both iCBT and gCBT are effective. iCBT was 1.7 times as cost-effective as the group treatment 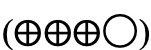
Kaldo et al [61], 2013	IG2: iCBT 6-step treatment program, each step consisting of 1-4 modules + interactive homework+ registration + reporting	Guidance by team of psychologists monitoring progress of patient	IG1: low-intensity iCBT	7-10 days/treatment step ≥6-9 weeks with extension possible if motivated	3 months	TRQ (primary outcome) HADS, ISI tinnitus distress (0-9) tinnitus loudness (0-9)	After treatment: IG1: 37% IG2: 37%. At 3 months FU: IG1: 37%, IG2: 46%	For both low-intensity iCBT and iCBT, significant decreases (*P*<.001) were found on all measurement points for all measures with small to medium effect size	iCBT can successfully be used in a regular clinical setting to reduce tinnitus distress 
Kleinstäuber et al [64], 2018	iCBT: 12 mandatory and 6 optional text modules	Guidance by psychologist weekly through secured web-based messaging system to encourage patients or give them advice	Group CBT: 10 weekly sessions of 90 minutes in groups of 5-12 patients by psychologist + weekly personal supervision	10 weeks	T2: 6 months FU T3: 1 year FU	THI (primary outcome): BFI-10^an^	T1—IG: 7.3%, CG: 7.5%, T2—IG: 17.1%, CG: 14%, T3—IG: 26.8%, CG: 16.3%	THI scores improved on average –11.47 points from pre to post in iCBT and –14.71 points when controlling for all 5 BFI-10 subscales	iCBT might be the preferred treatment choice for patients with tinnitus who are open for new experiences, can motivate themselves, and work autonomously 
Rheker et al [67], 2015	IG2: iCBT: 12 mandatory and 6 optional text modules	Guidance by psychologist whenever patient needed it through email, to answer questions, for encouragement	IG1: iCBT without support	10 weeks	T2: 1 year FU	THI (primary outcome), mini-TQ (primary outcome), PHQ-9, PATHEV^ao^	After treatment: IG1: 16.1% IG2: 8.9%, At 1-year FU: IG1: 42.9%, IG2: 28.6%	Both groups experienced reduced tinnitus distress through the study	iCBT is effective reducing tinnitus distress, even if no support is provided 
Weise et al [66], 2016	iCBT: 12 mandatory and 6 optional text modules	Communication through secured encrypted webpage with psychologist once a week (+ max 10-minute email/week)	Confidential, moderated, web-based discussion forum	10 weeks	T2: 6-month FU T3: 1-year FU	THI (primary outcome) mini-TQ, HADS, ISI, TAQ	T1: IG: 6.5%, CG: 1.6%, T2: IG: 6.5%, T3: IG: 11.3%	A reliable change was reached by 72.6% of IG regarding THI and 80.6% for mini-TQ	iCBT is effective in the treatment of severe, chronic tinnitus 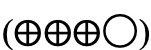

^a^FU: follow-up.

^b^GRADE: Grading of Recommendations Assessment, Development, and Evaluation.

^c^TIP: Tinnitus Information Program.

^d^MCQ: multiple choice question.

^e^N/A: not applicable.

^f^SITHC: Structural Interview for Tinnitus History and Complaints.

^g^CITD: Clinical Interview for Tinnitus Distress.

^h^TRQ: Tinnitus Reaction Questionnaire.

^i^DASS: Depression Anxiety Stress Scales.

^j^WHOQOL-BREF: World Health Organization Quality of Life.

^k^OSI-R: Occupational Stress Inventory-Revised.

^l^VAS: visual analog scale.

^m^QOS: quality of sleep.

^n^TCS: Tinnitus Catastrophizing Scale.

^o^TSQ: Tinnitus severity questionnaire.

^p^IG: intervention group.

^q^CG: control group.

^r^PSQ: Personal Strain Questionnaire.

^s^

: low certainty.

^t^TFI: Tinnitus Functional Index.

^u^GAD-7: Generalized Anxiety Disorder.

^v^PHQ-9: Patient Health Questionnaire.

^w^ISI: Insomnia Severity Index.

^x^TCQ: Tinnitus Cognitions Questionnaire.

^y^EQ-5D-5L: EuroQol-5 dimensions.

^z^THS: Tinnitus and Hearing Survey.

^aa^THI-S: Tinnitus Handicap Inventory-Short Form.

^ab^HHIA-S: Hearing Handicap Inventory for Adults-Short Form.

^ac^HQ: Hyperacusis Questionnaire.

^ad^CFQ: Cognitive Failures Questionnaire.

^ae^SWLS: Satisfaction with Life Scale.

^af^
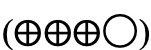
: moderate certainty.

^ag^iACT: internet-based acceptance and commitment therapy.

^ah^HADS: Hospital Anxiety and Depression Scale.

^ai^QoLI: Quality of Life Inventory.

^aj^PSS: Perceived Stress Scale.

^ak^TAQ: Tinnitus Acceptance Questionnaire.

^al^TQ: Tinnitus Questionnaire.

^am^gCBT: group cognitive behavioral therapy.

^an^BFI-10: Big Five Inventory.

^ao^PATHEV: Patient Questionnaire on Therapy Expectation and Therapy Evaluation.

**Table 2:** Table of evidence: internet-based cognitive behavioral therapy (iCBT) with guidance (noncontrolled trials).

Author, year	Intervention	Guidance	Duration intervention	FU^a^	Outcome measures	Dropout	Data results	Conclusion + GRADE^b^ score
Beukes et al [51], 2018	iCBT: 16 recommended modules + 5 optional modules + homework assignments + worksheets + quizzes	Guidance by audiologist: outlining content, monitoring progress, providing feedback, answering questions + encrypted 2-way message system within ePlatform	8 weeks (2 modules/week + 1 extra optional module each week between week 2 and 6)	1 year	TFI^c^ (primary outcome), GAD-7^d^, PHQ-9^e^, ISI^f^, HHIA-S^g^, HQ^h^, CFQ^i^, SWLS^j^, SSQ^k^	28.8%	TFI improved by 22.7 (+22.85 or –22.85) points between T0 and T3, with a clinically significant change for 46% of patients (Cohen *d*=1.04)	The benefits of audiologist guided iCBT are maintained 1-year after intervention 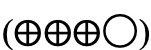 ^l^
Beukes et al [52], 2016	iCBT: 16 recommended modules + 5 optional modules + homework assignments + worksheets + quizzes	Guidance by audiologist: outlining content, monitoring progress, providing feedback, answering questions + encrypted 2-way message system within ePlatform	8 weeks (2 modules/week + 1 extra optional module each week between week 2 and 6)	N/A^m^	TFI (primary outcome) THI-S^n^, GAD-7, PHQ-9	21.6%	38% of participants reached clinically significant change of 23.86. Difference TFI pre-post intervention was statistically significant (Cohen *d*=1.18; t_36_=6.26; *P*<.001)	An internet-based intervention of tinnitus appears to be feasible in the United Kingdom when using audiological support 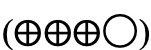
Beukes et al [57], 2021	iCBT: 22 modules with explanatory videos + homework assignments + worksheets + quizzes	On-demand support by audiologist: encrypted 2-way message system within ePlatform	8 weeks (2-3 modules/week)	2 months	TFI (primary outcome) THI-S, TQQ^o^, ASQ^p^	After treatment: 31.3%. At 2-month FU: 50%	A pre-post score difference of 19.3 on the TFI was considered clinically significant; this was obtained by 44% at both T1 and T2	iCBT for Spanish communities appears to be feasible 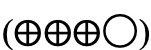
Beukes et al [57], 2021	iCBT: 22 modules with explanatory videos + homework assignments + worksheets + quizzes	Guidance by audiologist: introducing and outlining module content, monitoring progress, providing feedback on worksheets, answering questions + encrypted 2-way message system within ePlatform	8 weeks (2-3 modules/week)	2 months	TFI (primary outcome), THI-S, TQQ, GAD-7, PHQ-9, ISI, HHIA-S, HQ, CFQ, SWLS	After treatment: 14.8%. At 2-month FU: 33.3%	A pre-post score difference of 19.51 on the TFI was considered clinically significant, this was obtained by 70% (completer’s analysis)	iCBT was found to be feasible for tinnitus treatment in the United States 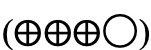
Kaldo et al [62], 2004	iCBT: 6-step D13:M13 treatment program, each step consisting of 1-4 modules + 16 mandatory tools + interactive homework + self-help material + worksheets + diary + reporting	Guidance by psychologists through email or telephone to give feedback, support, give recommendations, foster adherence, or help out with problems	6-10 weeks	3 months	TRQ^q^ (primary outcome), THI, HADS, ISI. Treatment credibility, therapist-patient interaction, patient compliance	After treatment: 30% at 3-month FU: 28% (without perfect overlap only 50.6% filled in both the posttreatment as the FU assessment)	A significant improvement (*P*<.001) was found for all measures at all time points	iCBT can be transferred into clinic, but dropout rates are critical. If fulfilled, the full program improvements are noted after treatment and maintained at 3-month FU 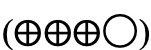
Probst et al [65], 2019	iCBT: 12 mandatory and 6 optional text modules	GUIDANCE by psychologist 10 minutes weekly through secured web-based messaging system to encourage patients or give them advice	10 weeks	N/A	THI (primary outcome), HADS, TAQ, ISI, ASI-3^r^, BFI-10^s^, WAI-SR^t^	6.8% without THI after assessment were considered as nonresponders	Responders significantly improved from baseline to both midtreatment and after treatment (completer and ITT^u^: *P*<.001)	No symptom change in the first half of iCBT for chronic tinnitus is a risk factor of not benefiting from iCBT 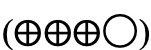

^a^FU: follow-up.

^b^GRADE: Grading of Recommendations Assessment, Development, and Evaluation.

^c^TFI: Tinnitus Functional Index.

^d^GAD-7: Generalized Anxiety Disorder.

^e^PHQ-9: Patient Health Questionnaire.

^f^ISI: Insomnia Severity Index.

^g^HHIA-S: Hearing Handicap Inventory for Adults-Short Form.

^h^HQ: Hyperacusis Questionnaire.

^i^CFQ: Cognitive Failures Questionnaire.

^j^SWLS: Satisfaction with Life Scale.

^k^SSQ: Speech, Spatial and Qualities of Hearing.

^l^
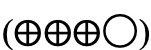
:moderate certainty.

^m^N/A: not applicable.

^n^THI-S: Tinnitus Handicap Inventory-Short Form.

^o^TQQ: Tinnitus Qualities Questionnaire.

^p^ASQ: Abbreviated Symptoms Questionnaire.

^q^TRQ: Tinnitus Reaction Questionnaire.

^r^ASI-3: Anxiety Sensitivity Index-3.

^s^BFI-10: Big Five Inventory.

^t^WAI-SR: Working Alliance Inventory-Short Revised.

^u^ITT: intent-to-treat.

### iCBT Without Guidance

#### Characteristics of Included Studies

Overall, 4 controlled trials [61,67-69] examined iCBT without guidance as a telerehabilitation intervention. All studies were published between 2002 and 2015. The total study sample included 940 participants, of which 278 (30%) fulfilled an iCBT program without guidance. Table S2 in Multimedia Appendix 3 provides detailed information of the included studies.

#### Results of Individual Studies

The iCBT program of Andersson et al [68] is based upon the principles of CBT by Hawton et al [80]. The other studies based the content of the iCBT program upon the principles of the self-help manual of Andersson and Kaldo [79]. Therefore, the content of the iCBT programs with and without guidance was similar. Variation in structure and duration of the iCBT program among the studies was noted. All 4 studies concluded that iCBT without guidance significantly reduced tinnitus distress and was therefore considered an effective alternative or additional treatment tool. The specifications of the studies are provided in Table 3.

Dropout rates for unguided iCBT ranged from 16.1% up to 37% after treatment and from 37% up to 42.9% at follow-up. It was more effective than wait-listing and as effective as group CBT or guided iCBT. All studies noted similar dropout rates for the intervention and the control group except for Rheker et al [67], who found that the dropout rates in the iCBT with guidance group were approximately only half of the dropout rates found for the iCBT without guidance group both after treatment and at follow-up. The most noted barrier for compliance in this case was lack of time [67] The dropout rates in iCBT with and without guidance groups after treatment are further illustrated in Table 4.

**Table 3:** Table of evidence: internet-based cognitive behavioral therapy (iCBT) without guidance (controlled trials).

Author, year	Intervention	Comparison	Duration intervention	FU^a^	Outcome measures	Dropout	Data results	Conclusion + GRADE^b^ score
Andersson et al [68], 2002	iCBT self-help manual: 10 components in 6 modules + homework assignments + weekly reports	Waiting list, received program later	6 weeks	1 week after treatment-controlled FU 1-year uncontrolled FU	TRQ^c^ (primary outcome), HADS^d^, ASI^e^, VAS^f^ tinnitus loudness, VAS tinnitus annoyance, VAS control over tinnitus, VAS quality of sleep	18.8%	Statistically significant change scores were found for all measures (*P*<.05), except for quality of sleep ratings (*P*=.10)	iCBT can be used as an adjunct treatment tool for tinnitus patients, but does need further development  ^g^
Kaldo et al [61], 2013	IG1^h^: low-intensity iCBT: patients receive all modules at once at get 3 months to read the most personally relevant modules by themselves	IG2: iCBT: 6-step treatment program, each step consisting of 1-4 modules + interactive homework+ registration + reporting	3 months	3 months	TRQ (primary outcome), HADS, ISI^i^, Tinnitus distress (0-9), Tinnitus loudness (0-9)	After treatment: IG1: 37%, IG2: 37% at 3 months FU: IG1: 37%, IG2: 46%	All measures at all measurement moments *P*<.001	iCBT can successfully be used in a regular clinical setting to reduce tinnitus distress, a low-intensity version can be used for patients with lower distress levels or motivation 
Nyenhuis et al [69], 2013	IG1: iCBT based on CBT manual (structure not further specified); IG2: bibliotherapy self-help manual CBT	IG3: group CBT based on CBT manual + therapeutic contact (not further specified) CG^j^: information	3 months	N/A^k^	TQ^l^	IG1: 34.48%, IG2: 34.48%, IG3: 34.62%, CG: 30.59%	Effect sizes when compared with control of IG1, IG2, and IG3 where: Cohen *d*=1.04, *d*=0.24, and *d*=0.89, respectively	All 3 efficaciously reduced tinnitus distress 
Rheker et al [67], 2015	IG1: 12 mandatory and 6 optional text modules	IG2: iCBT with on-demand support	10 weeks	T2: 1 year FU	THI^m^ (primary outcome), mini-TQ (primary outcome), PHQ-9^n^, PATHEV^o^	After treatment: IG1: 16.1%, IG2: 8.9%. At 1-year FU: IG1: 42.9%, IG2: 28.6%	No significant difference between both groups was found (χ^2^_2_=1.3; *P*=.52)	iCBT is effective in reducing tinnitus distress, even if no support is provided 

^a^FU: follow-up.

^b^GRADE: Grading of Recommendations Assessment, Development, and Evaluation.

^c^TRQ: Tinnitus Reaction Questionnaire.

^d^HADS: Hospital Anxiety and Depression Scale.

^e^ASI: Anxiety Sensitivity Index.

^f^VAS: visual analog scale.

^g^

: low certainty.

^h^IG: intervention group.

^i^ISI: Insomnia Severity Index.

^j^CG: control group.

^k^N/A: not applicable.

^l^TQ: Tinnitus Questionnaire.

^m^THI: Tinnitus Handicap Inventory.

^n^PHQ-9: Patient Health Questionnaire.

^o^PATHEV: Patient Questionnaire on Therapy Expectation and Therapy Evaluation.

**Table 4:** Dropout rates for internet-based cognitive behavioral therapy (iCBT) with guidance versus iCBT without guidance.

Study	Intervention group (%)	Control group (%)
Abbott et al [50], 2009	71.4	30.4
Beukes et al [55], 2021	52.4	55.6
Beukes et al [51], 2018	28.8	0
Beukes et al [52], 2016	21.6	0
Beukes et al [53], 2018	4.3	4.3
Beukes et al [56], 2017	14	1.4
Beukes et al [54], 2021	31	0
Beukes et al [57], 2021	14.8	0
Hesser et al [58], 2012	4	0
Jasper et al [59], 2014	7.3	7
Kaldo et al [62], 2004	30	0
Kaldo et al [63], 2008	4	0
Kaldo et al [61], 2013	37	37
Kleinstauber et al [64], 2018	7.3	7.5
Probst et al [65], 2019	6.8	0
Rheker et al [67], 2015	8.9	16.1
Weise et al [66], 2016	6.5	1.6
Andersson et al [68], 2002	18.8	NS^a^
Nyenhuis et al [69], 2013	34.48	30.59

^a^NS: not specified.

### Self-help Devices

#### Characteristics of Included Studies

Only 2 studies [70,71] performed research concerning the use of a self-help device as a telerehabilitation treatment form. Hyvärinen et al [70] examined the use of a self-administrable form of transcranial direct current stimulation (tDCS) for a sample of 43 participants with chronic tinnitus. The second study, Schlee et al [71], used an auricular acupressure device in combination with a self-help smartphone app as a treatment for a sample of 39 patients with chronic tinnitus. The details of both studies are presented in Table S3 in Multimedia Appendix 3.

#### Results of Individual Studies

Hyvärinen et al [70] concluded that THI scores significantly decreased (*P*<.05) between pre- and posttreatment assessments for all groups. This means patients receiving tDCS, in whichever application form, as well as most of the control group receiving sham tDCS, improved. Schlee et al [71], in contrast, concluded that the use of an acupressure device in conjunction with a self-help smartphone app caused a significant negative linear trend for both tinnitus loudness (*P*<.001; Cohen *d*=0.861) and tinnitus distress (*P*=.005; Cohen *d*=0.478). Table 5 provides detailed information of the included studies.

**Table 5:** Table of evidence: self-help devices.

Author, year	Intervention	Comparison	Duration intervention	Follow-up	Outcome measures	Dropout	Data results	Conclusion + GRADE^a^ score
Hyvärinen et al [70], 2016	At-home tDCS^b^: one in outpatient clinic session after a training and 9 at-home sessions (20 minute/session), IG1: left temporal area anodal, IG2: bifrontal, IG3: Sham	Standard in clinic tDCS	10 days	4 weeks	THI^c^ (primary outcome), TQ^d^, BDI-IA^e^, BAI^f^, VAS^g^ tinnitus loudness or annoyance; user experience questionnaire	18.6%	THI scores in the noncontrolled groups decreased significantly from before to after treatment (mean change –5.0; t_29_=–2.14; *P*<.05)	Self-administered at-home tDCS was safe and easy to use and gave similar results compared with in clinic trials.  ^h^
Schlee et al [71], 2021	Acupressure device + app giving weekly coping and self-help tips	NA^i^	6 weeks	N/A	TSCHQ^j,^ EMA^k^ through smartphone app	No dropout reported	Negative linear trends were found for both tinnitus loudness and tinnitus distress, respectively (*P*<.001; Cohen *d*=–0.861) and (*P*=.005; Cohen *d*=–0.478)	The use of an acupressure device in conjunction with a self-help app appeared to be effective as a tinnitus treatment 

^a^GRADE: Grading of Recommendations Assessment, Development, and Evaluation.

^b^tDCS: transcranial direct current stimulation.

^c^THI: Tinnitus Handicap Inventory.

^d^TQ: Tinnitus Questionnaire.

^e^BDI-IA: Beck Depression Inventory.

^f^BAI: Beck Anxiety Inventory.

^g^VAS: visual analog scale.

^h^

: low certainty.

^i^N/A: not applicable.

^j^TSCHQ: Tinnitus Sample Case History Questionnaire.

^k^EMA: ecological momentary assessment.

### Self-help Manuals

#### Characteristics of Included Studies

One RCT study [60,69] looked into the use of a self-help manual as a form of telerehabilitation. The study sample included 72 participants, of which 34 participants received the self-help manual as treatment. Table S4 in Multimedia Appendix 3 provides further details of this study.

#### Results of Individual Studies

The study used a self-help manual or book as bibliotherapy based upon the principles of CBT and guidance was provided for the participants in the form of weekly telephone calls with a psychologist. This intervention was compared with wait-listing. Control patients received the self-help book without guidance after a waiting period of 6 weeks. The study concluded, after a follow-up of 1 year, that a self-help manual can serve as a convenient and effective alternative way to administer CBT and significantly reduce tinnitus distress (Tinnitus Reaction Questionnaire: *P*≤.003; THI: *P*<.02). This study had a GRADE score of moderate certainty.

### Smartphone Apps

#### Characteristics of Included Studies

A total of 8 articles [71-78], with a combined sample size of 284 participants, discussed the use of a smartphone app as part of the treatment of patients with tinnitus. Clarification on the study and patient characteristics of the studies examining the use of a smartphone app are presented in Table S5 in Multimedia Appendix 3.

#### Results of Individual Studies

All studies used smartphone apps delivering a form of sound therapy for patients with tinnitus, except for the ones by Henry et al [75] and Schlee et al [71] (see the *Self-help Devices* section). Henry et al [75] delivered progressive tinnitus management through a smartphone app and showed favorable results, with 32% of the participants achieving a clinically meaningful reduction of 13 points on the TFI. In general, the sound therapy apps did show promising results. Abouzari et al [72] used an application that, in addition to the sound therapy, provided 8 CBT modules. This treatment showed potential and significantly greater improvement in THI than wait-listing. Barozzi et al [73] used streaming nature of hearing aids or standard broadband noise through connection with a smartphone app and concluded both the nature sounds and broadband noise sounds caused a significant decline (*P*<.001) in TFI scores. Chatterjee et al [74] and Kutyba et al [77] concluded that the ReSound Tinnitus Relief app delivering sound therapy also caused significant reductions (*P*<.001) in both THI and TFI scores. Tyler et al [78] allowed this ReSound Tinnitus Relief app to send sounds to cochlear implants, and all patients noted a lower visual analog scale after treatment with the app. The study by Kim et al [76] revealed that combined notched music therapy through a smartphone app with Gingko biloba treatment significantly (*P*=.03) lowered TFI scores after 3 months of treatment. Tables 6 and 7 provide further specifications.

**Table 6:** Table of evidence: smartphone apps (controlled trials).

Study	Intervention					Outcome			Results	
Author, year	Intervention	Comparison	Duration intervention	FU^a^	Outcome measures		Dropout	Data results		Conclusion + GRADE^b^ score
Abouzari et al [72], 2009	2 hours daily listening to sound therapy and 2-3 hours weekly on 8 CBT^c^ modules provided through smartphone app	Waiting list	8 weeks	N/A^d^	THI^e^, GAD-7^f^, PSS^g^		No dropout reported	IG^h^ reported a significantly greater improvement in THI scores (*P*=.04)		This pilot study showed a potentially promising efficacy of a smartphone-based CBT and sound therapy platform for treating tinnitus  ^i^
Barozzi et al [73], 2021	One counseling session and the use of hearing aids streaming nature sounds through connection with smartphone app (8 hours/day)	One counseling session and the use of hearing aids streaming standard BB^j^ noise (8 hours/day)	6 months	FU1: 3 months after fitting; FU2: 6 months after fitting	THI, NRS^k^		No dropout reported	A significant (*P*<.001) mean decline of 10.96 points (95% CI 8.08-13.83) was observed for both time periods BL^l^ to FU1 and FU1 to FU2 in both groups		Significant reductions of TFI^m^ were found for both groups; neither appeared to be superior ⨁⨁◯◯
Chattarjee et al [74], 2020	Sound therapy by ReSound Tinnitus Relief app + 8 weekly counseling sessions	MBTSR^n^ through video call (8 weekly 2 hours sessions) + 60 daily sessions of MBTSR home exercises	2 months	1 month after treatment	PATARR^o^, TCQ^p^, THI		No dropout reported	Significant reduction of TFI and TCQ (*P*<.001) between pre- and posttreatment outcome measure results		Both home-based management was successful both in form of MBTSR as sound therapy through the ReSound Tinnitus Relief app 

^a^FU: follow-up.

^b^GRADE: Grading of Recommendations Assessment, Development, and Evaluation.

^c^CBT: cognitive behavioral therapy.

^d^N/A: not applicable.

^e^THI: Tinnitus Handicap Inventory.

^f^GAD-7: Generalized Anxiety Disorder.

^g^PSS: Psychiatric Signs Screening.

^h^IG: intervention group.

^i^

: low certainty.

^j^BB: broadband.

^k^NRS: Numeric Rating Scale.

^l^BL: baseline.

^m^TFI: Tinnitus Functional Index.

^n^MBTSR: mindfulness-based tinnitus stress reduction.

^o^PATARR: Personalized Android-based Tinnitus Apps Resound Relief.

^p^TCQ: Tinnitus Cognitions Questionnaire.

**Table 7:** Table of evidence study smartphone apps noncontrolled trial.

Study	Intervention				Outcome			Results	
Author, year	Intervention	Duration intervention	FU^a^	Outcome measures		Dropout	Data results		Conclusion + GRADE^b^ score
Henry et al [75], 2017	PTM^c^ through a smartphone app	6-8 weeks	N/A^d^	TFI^e^		8%	32% of patients achieved a meaningful reduction of ≥13 points on the TFI		The concept that a PTM app might be successful was proven; this new approach still needs to be tested further  ^f^
Kim et al [76], 2016	Notched music therapy through a smartphone app (30-60 minutes) in combination with Ginko treatment	3 months	N/A	THI^g^, VAS^h^ tinnitus loudness, noticeable time, annoyance, or disruption of daily life, BDI^i^, STAI^j^, PSQI^k^		No dropout reported	The THI score was significantly lower after 3 months of treatment with the notched music therapy app and Ginko (*P*=.03)		The use of a smartphone-delivered notched music therapy in combination with Ginko appeared to be effective in reducing tinnitus distress 
Kutyba et al [77], 2022	Sound therapy by ReSound Tinnitus Relief app (30 min/day)	6 months	FU1: 3 months, FU2: 6 months	TFI, THI		No dropout reported	Both THI and TFI decreased significantly from BL to FU1 and FU2 (*P*<.002)		The use of the ReSound Tinnitus Relief app may contribute to tinnitus reduction 
Schlee et al [71], 2021	Acupressure device + app giving weekly coping and self-help tips	6 weeks	N/A	TSCHQ^l^, EMA^m^ through smartphone app		No dropout reported	Negative linear trends were found for both tinnitus loudness and tinnitus distress, respectively (*P*<.001; Cohen *d*=–0.861) and (*P*=.005; Cohen *d*=–0.478)		The use of an acupressure device in conjunction with a self-help app appeared to be effective as a tinnitus treatment 
Tyler et al [78], 2018	Sound therapy by ReSound Tinnitus Relief app sending sounds to CI^n^	2 weeks	N/A	Tinnitus loudness and effectiveness app rating from 0-100		23.1%	3 out of 10 participants rated the effectiveness ≥70%		The use of an app-delivered sound therapy sending sounds to CI can be acceptable and might relief some patients with tinnitus 

^a^FU: follow-up.

^b^GRADE: Grading of Recommendations Assessment, Development, and Evaluation.

^c^PTM: progressive tinnitus management.

^d^N/A: not applicable.

^e^TFI: Tinnitus Functional Index.

^f^

: low certainty.

^g^THI: Tinnitus Handicap Inventory.

^h^VAS: visual analog scale.

^i^BDI: Beck Depression Inventory.

^j^STAI: State Trait Anxiety Inventory.

^k^PSQI: Pittsburg Sleep Quality Index.

^l^TSCHQ: Tinnitus Sample Case History Questionnaire.

^m^EMA: ecological momentary assessment.

^n^CI: Cochlear Implant.

### Other Internet-Based Interventions

#### Characteristics of Included Studies

Overall, 2 studies [58,74], which are also discussed in previous categories, had an additional intervention group receiving a treatment form not allocable to one of the subcategories above. Chattarjee et al [74] compared the ReSound Tinnitus Relief app and an internet-based program of mindfulness-based tinnitus stress reduction. Both intervention groups included 30 participants. Hesser et al [58] examined an internet-based acceptance and commitment therapy (iACT) besides iCBT. A total of 35 participants with chronic tinnitus received ACT. Table S6 in Multimedia Appendix 3 provides detailed information of these included studies.

#### Results of Individual Studies

Chattarjee et al [74] concluded that mindfulness-based tinnitus stress reduction was effective in significantly (*P*<.001) reducing TFI and Tinnitus Cognitions Questionnaire and showed results comparable with the ReSound Tinnitus Relief app. Hesser et al [58] concluded that iCBT and iACT were both equally effective in reducing THI scores and might form opportunities to improve access to psychological interventions for tinnitus. Table 8 provides further information of the included studies.

**Table 8:** Table of evidence: other internet-based interventions (controlled trials).

Author, year	Intervention	Guidance	Comparison	Duration intervention	FU^a^	Outcome measures	Dropout	Data results	Conclusion + GRADE^b^ score
Chattarjee et al [74], 2020	IG2^c^: MBTSR^d^ through video call (8 weekly 2-hour sessions) + 60 daily sessions of MBTSR home exercises	Reporting once a week + 8 weekly counseling sessions	IG1: Sound therapy by ReSound Tinnitus Relief app	2 months	1 month after treatment	PATARR^e^, TCQ^f^, THI^g^	No dropout reported	Significant reduction of TFI^h^ and TCQ (*P*<.001) between pre- and posttreatment outcome measure results	Home-based management was successful in form of MBTSR as sound therapy through the ReSound Tinnitus Relief app  ^i^
Hesser et al [58], 2012	IG1: iCBT^j^—a self-help manual divided into 8 modules + exercises and homework assignments; IG2: internet-based ACT^k^—a self-help manual divided into 8 modules+ exercises and homework assignments	Feedback provided by psychologist on homework assignments through contact handling system + encouragement	CG^l^: confidential moderated discussion forum specifically targeting tinnitus-related problems	8 weeks	1 year	THI (primary outcome), HADS^m^, ISI^n^, QoLI^o^, PSS^p^, TAQ^q^	In total: 4% after treatment and 6% at FU	Within-group effects were substantial before treatment and through 1 year FU for both treatments (95% CI –44.65 to 20.45]; Cohen *d*=1.34), with no significant difference between treatments (95% CI –14.87 to 11.21; Cohen *d*=0.16)	iCBT and iACT are both equally effective and might improve access to psychological interventions for tinnitus 

^a^FU: follow-up.

^b^GRADE: Grading of Recommendations Assessment, Development, and Evaluation.

^c^IG: intervention group.

^d^MBTSR: mindfulness-based tinnitus stress reduction.

^e^PATARR: Personalized Android-based Tinnitus Apps Resound Relief.

^f^TCQ: Tinnitus Cognitions Questionnaire.

^g^THI: Tinnitus Handicap Inventory.

^h^TFI: Hospital Anxiety and Depression Scale.

^i^

: low certainty.

^j^iCBT: internet-based cognitive behavioral therapy.

^k^ACT: acceptance and commitment therapy.

^l^CG: control group.

^m^HADS: Hospital Anxiety and Depression Scale.

^n^ISI: Insomnia Severity Index.

^o^QoLI: Quality of Life Inventory.

^p^PSS: Perceived Stress Scale.

^q^TAQ: Tinnitus Acceptance Questionnaire.

## Discussion

### Principal Findings

This systematic review aimed to provide an overview of the forms of telerehabilitation available for patients with tinnitus and the effectiveness of these forms. Telerehabilitation might be of great importance to promote the self-management of patients and improve the cost-effectiveness of the treatment [40,43,44]. Currently, the recommended treatments for patients with tinnitus are TRT and CBT, which are tailored to the needs of the patient [16,17]. Although these in-person treatment forms are proven to be effective, they are also time consuming and costly [25-27]. Telerehabilitation might, therefore, form a solution as a replacement of or an aid to the in-person care currently delivered [40,43,44]. In total, 29 articles, that dove into the subject of telerehabilitation, were found eligible for this systematic review. Some of these articles examined multiple telerehabilitation forms [50-78]. Overall, 6 categories of telerehabilitation could be differentiated, namely iCBT with guidance, iCBT without guidance, self-help devices, self-help manuals, smartphone apps, and other internet-based interventions. For every category, evidence was provided stating that this form of telerehabilitation was effective in reducing tinnitus distress and complaints [50-78].

As CBT is one of the most recommended evidence-based treatments for patients with tinnitus, it is not unusual that most studies took a closer look at iCBT. iCBT has proven to be effective for the treatment of posttraumatic stress disorder, anxiety, and depression [81-83]. The effectiveness of iCBT with guidance was also affirmed for the tinnitus treatment by 17 studies [50-67]. Most often, CBT is offered by a trained psychologist; however, in the 7 studies from the research group of Beukes [51-57], patients received guidance offered by an audiologist [6,21-23]. An interesting limitation of these studies concerning iCBT was the high dropout rates. Dropout from iCBT was also a concern when it was researched in the treatment of populations other than patients with tinnitus [81-83]. It is hard to differentiate whether patients did not complete follow-up assessments because they did not achieve the expected results and dropped out or because they did improve and did not find it necessary to receive further help and examination. Therefore, it is not certain that, in general, iCBT with guidance will be as beneficial as predicted for every patient. The high dropout rates for iCBT with guidance group forecast the same for iCBT without guidance group. This is because patients have to work autonomously to fulfill the modules of the iCBT course and do not receive motivational messages, feedback, support, or any form of additional guidance.

The current understanding of predictors of dropout is limited [83]. An important point of discussion that needs to be highlighted is that, according to previous research and clinical experience, CBT is a treatment form that might be susceptible for dropout by nature even as in-person care [84]. Further research is needed to assess why adherence and retention rates are low in iCBT and whether the reasons for dropout in in-person CBT are equivalent. Some of the included studies offered hypotheses for the high dropout rates. First of all, iCBT might not be engaging enough despite regular therapeutic encouragement [50,52]. In addition, it is highly demanding and time consuming for the patients [50,54,62]. Patients with lower levels of initial tinnitus distress appeared to be less motivated and more likely to drop out or be a nonresponder [50,51,55,65]. Finally, the attitude of the patient toward the treatment and the personality of the patient might be of great influence. Patients with an open personality, with a hope of improvement, and who believed the treatment was credible did show the best results [61,63,64,67]. It needs to be noted, however, that patients expecting positive results of treatment are often also patients who are likely to improve. Future iCBT interventions should address the barriers of lack of time, motivation, and engagement and should emphasize the link between efforts and results to the patient. The iCBT content has to be more engaging and should consist only of the most essential modules to limit the time investment. Options that need further exploration are, for example, increasing the interactivity, enhancing visual education material rather than plain text, implementing rewards within the program, or other ideas to increase compliance.

Internet-based intervention programs, such as iCBT, as well as others such as iACT, have been of great interest since the past decennia owing to the great advancements made in technology and the accessibility of the internet. Although iCBT and other CBT-based self-help treatments are a frequently researched topic in the world of telerehabilitation, the use of self-help devices is not a topic of research. In addition, the evidence for existing in practice therapies that are in need of a self-help device is limited compared with the evidence available concerning the effectiveness of CBT. In this review, only 2 studies with a small sample size examined the use of a self-help device. However, self-help devices form a window of opportunity to reduce the need for in-person care in treatment forms where several sessions are required to possibly achieve results. A risk involved with self-help devices is that not all patients are disciplined enough to perform the required treatment sessions at the requested time. For these categories of telerehabilitation through other internet-based interventions, the dropout rates for self-help manuals and self-help devices were most often not reported. Smartphone apps are more practical and portable than self-help devices. Almost every adult uses a smartphone in daily life. How convenient would it be if patients with tinnitus could use the device they already keep on them, their smartphone, as a treatment tool? Not only in the field of tinnitus, but in medicine and rehabilitation in general, the use of smartphone apps to offer treatment from a distance is on the rise and forms the newest trending topic in the field of medicine [85,86]. The same evolution is observed in the field of audiology. In the past years, the number of smartphone apps developed has increased widely. Some of these apps target the treatment of tinnitus, most often in the form of sound therapy [42,43]. Sound therapy, delivered through a smartphone app, appeared to be effective in reducing tinnitus distress. An important note, however, is that the smartphone apps were only tested in smaller sample sizes. Although the previous forms of telerehabilitation observed high dropout rates, the dropout rates for the use of smartphone apps were not reported, except for 2 studies. Henry et al [75] reported a dropout rate of 8% and Tyler et al [78] reported a dropout rate of 23.1%.

However, there are some limitations for the studies included in this systematic review. All included studies scored some to high concerns of RoB. As a result, the included studies are of low to moderate certainty. Furthermore, a comparison of effectiveness among studies was not feasible owing to the variety of questionnaires used to measure the level of tinnitus complaints, severity, or distress. In addition, some crucial confounding parameters were not considered by all studies. Only a minority of studies concerning iCBT in this review observed the proportion of participants with hearing loss or how many patients used hearing aids. However, how these studies define the presence of hearing loss was often not clarified. The studies concerning other telerehabilitation forms such as self-help manuals, self-help devices, and smartphone apps did seldomly define the percentage of participants with hearing loss. Hearing loss is an important risk factor for developing tinnitus [1]. In addition, there are patients with audiometrically normal hearing who experience tinnitus [1,87,88], but tinnitus severity and distress are known to be significantly worse in patients with tinnitus with hearing loss in comparison with patients with tinnitus without hearing loss [89]. Therefore, hearing loss might be an important confounding variable to consider. An audiometric examination of patients with tinnitus is therefore of value when researching patients with tinnitus.

Although hearing loss is an important confounding factor to keep in mind, other factors also need to be considered. First, gender plays a role in how patients perceive their tinnitus [90-95]. Men show less tinnitus-related distress compared with women [90-92]. Consequently, they might react differently to treatment [94]. Second, the prevalence of chronic tinnitus increases with age and older patients scored the loudness, annoyance, and distress of the experienced tinnitus higher [96,97]. As a consequence, the age of the patient might have an influence on how beneficial a treatment is. Third, the mental state of the patient, which can be measured using, for example, the Hospital Anxiety and Depression Scale, might be important. Tinnitus is known to be closely associated with anxiety, depression, stress, sleeping problems, etc [95,98,99]. The more serious these comorbidities are, the worse is the prognosis of the tinnitus [99]. These additional complaints severely influence the choice of the therapeutic plan [95,98,99]. Subsequently, hyperacusis is often a comorbid complaint of tinnitus patients, resulting in higher tinnitus severity and mental distress [100]. Finally, some patients might have neck or jaw complaint and this somatosensory afference might influence their tinnitus. This is referred to as somatic tinnitus or somatosensory tinnitus, often characterized by simultaneous onset or increase and decrease of tinnitus and the neck or jaw complaints [101,102]. The presence of somatic tinnitus requires an adapted treatment approach. Physiotherapy can be used to reduce the neck or jaw complaints and can consequently alleviate the tinnitus [103]. All the factors mentioned earlier might have an impact on the effectiveness of a treatment and should therefore be considered in future research.

### Conclusions

The results of this systematic review indicate that overall, there is low to moderate quality evidence that telerehabilitation in the form of iCBT with or without guidance, self-help manuals, self-help devices, smartphone apps, and other internet-based interventions effectively reduces tinnitus severity and distress. Telerehabilitation might form an alternative or additional tool to the recommended in-person care that patients currently receive. This review accentuated, however, that the greatest barrier to the success of telerehabilitation is the lack of compliance to treatment. Factors such as lack of time, engagement, motivation, and openness of the patient resulted in participant dropout and should be considered. In addition, it was noted that all included studies showed some to high concerns of RoB resulting in low to moderate certainty of the statements concerning the effectiveness of the telerehabilitation treatment forms. Future research should consider limiting the RoB and should further explore which factors are most likely to cause the lack of compliance and how clinicians can counteract these factors. Owing to the advances in technology made every day, telerehabilitation will keep evolving and therefore remains a trending topic to follow-up.

## Appendix

Multimedia Appendix 1 Search strategy.

Multimedia Appendix 2 Risk of bias summarization.

Multimedia Appendix 3 Tables of study and patient characteristics.

## Abbreviations

CBT: cognitive behavioral therapy
GRADE: Grading of Recommendations Assessment, Development, and Evaluation
iACT: internet-based acceptance and commitment therapy
iCBT: internet-based cognitive behavioral therapy
PRISMA: Preferred Reporting Items for Systematic Reviews and Meta-Analyses
PROSPERO: International Prospective Register of Systematic Reviews
RCT: randomized controlled trial
RoB: risk of bias
tDCS: transcranial direct current stimulation
TFI: Tinnitus Functional Index
THI: Tinnitus Handicap Inventory
TRT: tinnitus retraining therapy
