# Supplementary material for: Effectiveness of Telerehabilitation Interventions for Self-management of Tinnitus: Systematic Review
Source: J Med Internet Res. 2023 Feb 9;25:e39076. doi: 10.2196/39076 (PMC9951082; doi:10.2196/39076)
Supplement: Multimedia Appendix 1 [file jmir_v25i1e39076_app1.doc]

**Multimedia Appendix 1.** Search strategy.

| **Database** | **Search string** | **articles** |
| --- | --- | --- |
| **Pubmed** | Tinnitus [MeSH Terms] AND (("internet based intervention"[MeSH Terms]) OR (self care[MeSH Terms]) OR (self management[MeSH Terms]) OR ("mobile applications"[MeSH Terms]) OR ("telerehabilitation"[MeSH Terms]) OR (smartphone[MeSH Terms]) OR (Telemedicine [MeSH Terms]) OR Telehealth OR Telemedicine OR smartphone OR telerehab* OR “mobile application” OR “self manag*” OR “self care” OR “internet based intervention") | 74 |
| **Web of science** | TS=(self NEAR/3 (care OR management OR help) OR mobile applications OR internet based intervention OR web based intervention OR online intervention OR internet intervention OR telerehabilitation OR smartphone OR telemedicine OR telehealth OR mobile apps OR software apps OR virtual rehabilitation) AND TS=(tinnitus) | 266 |
| **Science Direct** | Tinnitus AND Smartphone application | 51 |
| **Cochrane Library** | *("self care" OR "self-care" OR "self management" OR "self-management" OR "self-help" OR "self help" OR "mobile applications" OR "internet based intervention" OR "internet-based intervention" OR "web based intervention" OR "web-based intervention" OR "online intervention" OR "internet intervention" OR "telerehabilitation" OR "tele-rehabilitation" OR smartphone OR telemedicine or tele-medicine OR telehealth OR tele-health OR "mobile apps" OR "software apps" OR "virtual rehabilitation") AND (tinnitus) in All Text | 56 |
| **Scopus** | ALL ( self  W/3  ( care  OR  management  OR  help )  OR  ( "mobile applications" )  OR  ( "internet-based intervention" )  OR  ( "web-based intervention" )  OR  ( "online intervention" )  OR  ( "internet intervention" )  OR  telerehabilitation  OR  telemedicine  OR  ( "mobile apps" )  OR  ( "software apps" )  OR  ( "virtual rehabilitation" ) )  AND  ALL ( tinnitus ) | 146 |
| **Total** | | 555 |
