# Supplementary figures and images for: Effectiveness of Telerehabilitation Interventions for Self-management of Tinnitus: Systematic Review
Source: J Med Internet Res. 2023 Feb 9;25:e39076. doi: 10.2196/39076 (PMC9951082; doi:10.2196/39076)

***
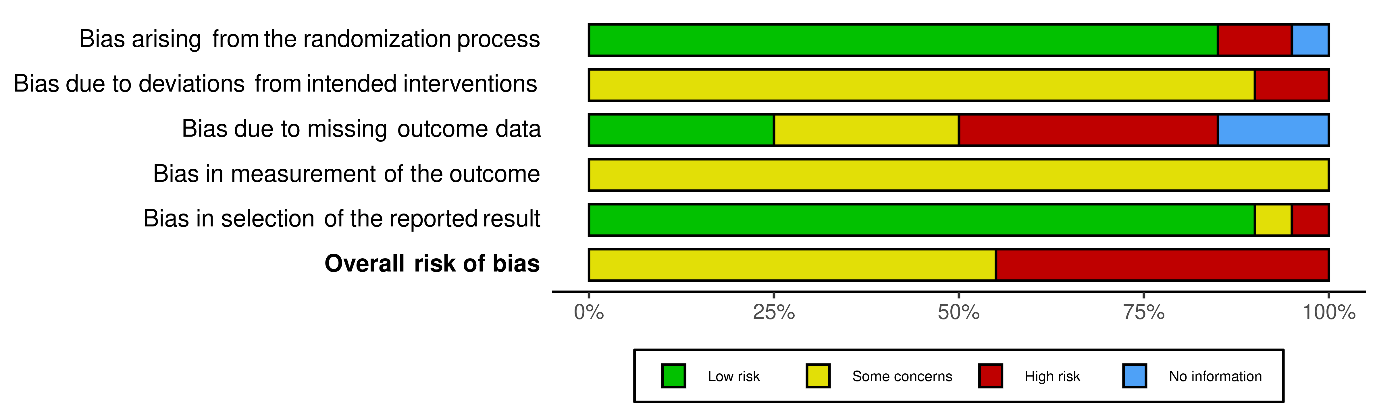
***

***Figure S1: Summary RoB controlled trials.***

***
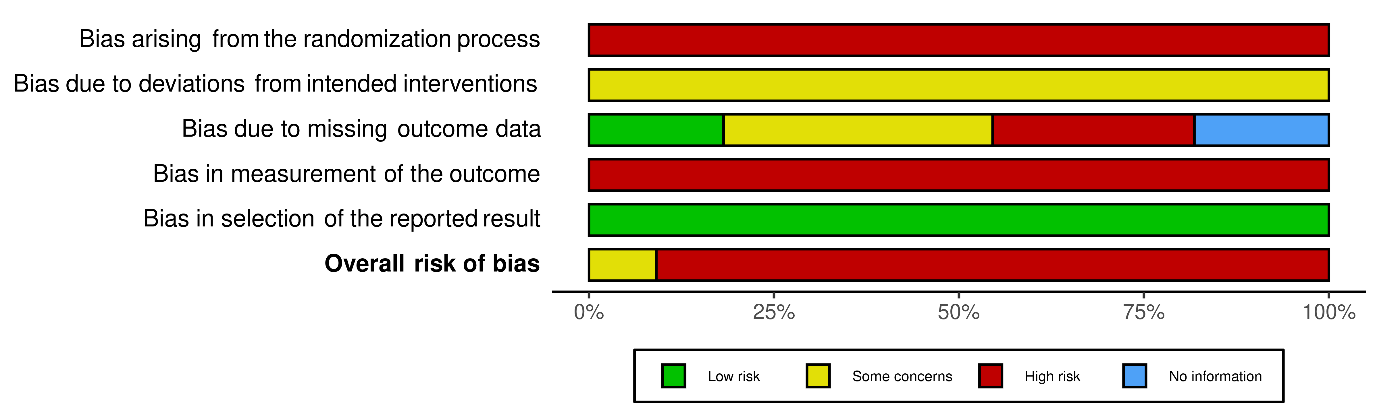
***

***Figure S2: Summary RoB non-controlled trials.***

Supplement: Multimedia Appendix 2 [file jmir_v25i1e39076_app2.docx]
