# Supplementary material for: Effectiveness of Telerehabilitation Interventions for Self-management of Tinnitus: Systematic Review
Source: J Med Internet Res. 2023 Feb 9;25:e39076. doi: 10.2196/39076 (PMC9951082; doi:10.2196/39076)
Supplement: Multimedia Appendix 3 [file jmir_v25i1e39076_app3.docx]

| ***Table S1: Study and population characteristics studies internet-based Cognitive Behavioral Therapy (iCBT) with guidance*** | | | | | | | | | | | | | | | | | |
| --- | --- | --- | --- | --- | --- | --- | --- | --- | --- | --- | --- | --- | --- | --- | --- | --- | --- |
| **Study characteristics** | | | | | | **Population characteristics** | | | | | | | | | | | |
| **Author** | **Date** | | | **Design** | | **Sample size** | | **Gender (%)** | | **Mean age (SD) (years)** | | **Type of tinnitus** | | **mean duration of tinnitus (SD) (years)** | | **Presence hearing loss (%)** | |
| ***Abbott***  ***et al.*** | 2009 | | | RCT | | IG: n=28 CG: n=23 | | IG:♂: 96% ♀: 4%  CG: ♂: 82% ♀: 18% | | IG: 50.5 (+/- 9.5)  CG: 48.7 (+/- 8.6) | | Chronic tinnitus (duration > 3 months) | | IG: 11.7 (+/- 9.6) CG: 5.0 (+/- 4.5) | | IG: 89% CG: 78% | |
| ***Beukes***  ***et al.*** | 2021 | | | RCT | | IG: n=63 CG: n=63 | | IG: ♂: 41% ♀: 59%  CG: ♂: 60% ♀: 40% | | IG: 55 (+/- 13)  CG: 57 (+/- 13) | | Chronic tinnitus (duration > 3 months) | | IG: 10 (+/- 11) CG: 15 (+/- 14) | | NS | |
| ***Beukes***  ***et al.*** | Nov  2018 | | | SG  non-CT | | n=104 | | ♂: 56% ♀: 44% | | 58.3 (+/- 12.5) | | Chronic tinnitus (duration > 3 months) | | 12 (+/-10.7) | | Use hearing aids: 36% | |
| ***Beukes***  ***et al.*** | 2016 | | | SG  non-CT | | n=37 | | ♂: 48.6% ♀: 51.4% | | 50-59 (+/- 1.32) | | Subjective chronic tinnitus | | 3-12 months: 10.8% 1-5 years: 43.2% 5-10 years: 16.2% 10+ years: 29.7% | | hearing loss: 70.3% use hearing aids: 27% | |
| ***Beukes***  ***et al.*** | Oct  2018 | | | SG  non-CT | | IG: n=46 CG: n=46 | | IG: ♂: 63% ♀: 37%  CG: ♂: 57% ♀: 43% | | IG: 50.7 (+/- 12.2) CG: 55.3 (+/- 11.6) | | Subjective chronic tinnitus | | IG: 5.23 (+/- 9.01) CG: 7.85 (+/- 9.62) | | Use hearing aids: IG: 41% CG: 41% | |
| ***Beukes***  ***et al.*** | 2017 | | | RCT | | IG: n=73 CG: n=73 | | IG: ♂: 59% ♀: 41%  CG: ♂: 55% ♀: 45% | | IG: 56.8 (+/- 12.2) CG: 54.3 (+/- 13.5) | | Chronic tinnitus (duration > 3 months) | | IG: 11.1 (+/- 11.5) CG: 12.4 (+/- 12.2) | | Use hearing aids: IG: 37% CG: 37% | |
| ***Beukes***  ***et al.*** | Sept  2021 | | | SG  non-CT | | n=32 | | ♂: 47% ♀: 53% | | 46.6 (+/- 11.0) | | Chronic tinnitus (duration > 3 months) with TFI>25 | | 6.0 (+/-8.09) | | NS | |
| ***Beukes***  ***et al.*** | August 2021 | | | SG  non-CT | | n=27 | | ♂: 33% ♀: 67% | | 55.48 (+/- 9.9) | | Subjective chronic tinnitus | | 11.75 (+/-13.36) | | Use hearing aids: 37% | |
| ***Hesser***  ***et al.*** | 2012 | | | RCT | | IG 1: n=32 IG 2: n=35 CG: n=32 | | IG 1: ♂: 56.2% ♀: 43.8%  IG 2: ♂: 57.1% ♀: 42.9%  CG: ♂: 56.2% ♀: 43.8% | | IG 1: 48.8 (+/- 13.4)  IG 2: 50.1 (+/-16.4)  CG: 48.4(+/- 14.2) | | Chronic tinnitus (duration > 6 months) with TFI>38 | | NS | | NS | |
| ***Jasper***  ***et al.*** | 2014 | | | RCT | | IG: n=41 CG 1: n=43 CG 2: n=44 | | IG 1: ♂: 61% ♀: 39%  IG 2: ♂: 55.8% ♀: 44.2%  CG: ♂: 63.6% ♀: 36.4% | | IG : 53.3 (+/- 9.8) CG 1: 50.2 (+/-13.1)  CG 2: 52.1 (+/- 9.0) | | Chronic tinnitus (duration > 6 months) with TFI>18 or mini THI>8 | | IG : 9.2 (+/- 7.9) CG 1: 8.4 (+/- 6.9) CG 2: 8.0 (+/- 7.1) | | IG : 68.3% CG 1: 72.1% CG 2: 77.3% | |
| ***Kaldo***  ***et al.*** | 2004 | | | SG  non-CT | | n=77 | | ♂: 52% ♀: 48% | | 46.1 (+/- 13.8) | | NS | | 7.0 (+/-8.0) | | Use hearing aids: 10% | |
| ***Kaldo***  ***et al.*** | 2008 | | | RCT | | IG: n=26 CG: n=25 | | IG: ♂: 58% ♀: 42%  CG: ♂: 56% ♀: 44% | | IG: 47.4 (+/- 12.9) CG: 45.0 (+/- 12.8) | | Chronic tinnitus (duration > 3 months) with TRQ>10 | | IG: 9.9 (+/- 13.5) CG: 5.6 (+/- 6.1) | | IG: 72% CG: 72% | |
| ***Kaldo***  ***et al.*** | 2013 | | | CT | | IG1: n=81 IG2: n=293 | | IG1: ♂: 67% ♀: 33%  IG2: ♂: 64% ♀: 36% | | IG1: 47.1 (+/- 14.1) IG2: 47.4 (+/- 13.6) | | Chronic tinnitus (duration > 3 months) | | IG1: 6.8 (+/- 8.0) IG2: 8.4 (+/- 10.8) | | IG1: 68% IG2: 68% | |
| ***Klein-***  ***stauber***  ***et al.*** | 2018 | | | RCT | | IG: n=41 CG: n=43 | | IG: ♂: 61% ♀: 39%  CG: ♂: 55.8% ♀: 44.2% | | IG: 51.32 (+/- 9.78) CG: 50.23 (+/- 13.13) | | Chronic tinnitus (duration > 6 months) with TFI>18 or mini THI>8 | | IG: 9.24 (+/- 7.88) CG: 8.35 (+/- 6.85) | | NS | |
| ***Probst***  ***et al.*** | 2018 | | | SG  non-CT | | n=103 | | ♂: 56.3% ♀: 43.7% | | 49.2(+/- 11.42) | | Chronic tinnitus | | NS | | NS | |
| ***Rheker***  ***et al.*** | 2015 | | | RCT | | IG1: n=56 IG2: n=56 | | IG1: ♂: 62.5% ♀: 37.5%  IG2: ♂: 62.5% ♀: 37.5% | | IG1: 54.14 (+/- 12.63)  IG2: 51.09(+/- 11.2) | | Chronic tinnitus (duration > 6 months) with TFI>18 or mini THI>8 | | IG1: 12.51 (+/- 12.86) IG2: 9.54 (+/- 9.23) | | IG1: 81.4% IG2: 74.2% | |
| ***Weise***  ***et al.***  RCT=Randomized Controlled Trial; non- CT= non-Controlled Trial; IG=Intervention Group; CG= Control Group; SD= Standard Deviation; NS= Not Specified; TFI= Tinnitus Functional Index | 2016 | | | RCT | | IG: n=62 CG: n=62 | | IG: ♂: 40.3% ♀: 59.7%  CG: ♂: 40.3% ♀: 59.7% | | IG: 47.81 (+/- 12.26) CG: 47.51 (+/- 14.07) | | Chronic tinnitus (duration > 6 months) with TFI>38 or mini THI>13 | | IG: 7.28 (+/- 6.81) CG: 7.29 (+/- 9.28) | | IG: 74.2% CG: 69.4% | |
| ***Table S2: Study and population characteristics studies internet-based Cognitive Behavioral Therapy (iCBT) without guidance*** | | | | | | | | | | | | | | | | |  |
| **Study characteristics** | | | | | **Population characteristics** | | | | | | | | | | | |  |
| **Author** | | **Date** | **Design** | | **Sample size** | | **Gender (%)** | | **Mean age (SD) (years)** | | **Type of tinnitus** | | **mean duration of tinnitus (SD) (years)** | | **Presence hearing loss (%)** | |  |
| ***Andersson***  ***et al.*** | | 2002 | RCT | | IG: n=53 CG: n=64 | | IG:♂: 54% ♀: 46%  CG:♂: 52% ♀: 48% | | IG: 48.5 (+/- 12.3) CG: 47.2 (+/- 15.0) | | Chronic tinnitus (duration > 6 months) | | IG: 6.2 (+/- 5.6) CG: 6.4 (+/- 6.8) | | IG: 69% CG: 68% | |  |
| ***Kaldo***  ***et al.*** | | 2013 | RCT | | IG1: n=81 IG2: n=293 | | IG1: ♂: 67% ♀: 33% IG2: ♂: 64% ♀: 36% | | IG1: 47.1 (+/- 14.1) IG2: 47.4 (+/- 13.6) | | Chronic tinnitus (duration > 3 months) | | IG1: 6.8 (+/- 8.0) IG2: 8.4 (+/- 10.8) | | IG1: 68% IG2: 68% | |  |
| ***Nyenhuis***  ***et al.*** | | 2013 | RCT | | IG 1: n=87 IG 2: n=87 IG 3: n=78 CG: n=85 | | ♂: 56.3% ♀: 43.7%  NS for each group | | 48.4 (+/- 12.6) | | Acute tinnitus (duration < 6 months) | | 0.27 (+/- 0.16) | | NS | |  |
| ***Rheker et al.*** | | 2015 | RCT | | IG1: n=56 IG2: n=56 | | IG1: ♂: 62.5% ♀: 37.5% IG2: ♂: 62.5% ♀: 37.5% | | IG1: 54.14 (+/- 12.63) IG2: 51.09(+/- 11.2) | | Chronic tinnitus (duration > 6 months) with TFI>18 or mini THI>8 | | IG1: 12.51 (+/- 12.86) IG2: 9.54 (+/- 9.23) | | IG1: 81.4% IG2: 74.2% | |  |

RCT=Randomized Controlled Trial; IG=Intervention Group; CG= Control Group; SD= Standard Deviation; NS= Not Specified; TFI= Tinnitus Functional Index

| ***Table S3: Study and population characteristics studies Self-help devices*** | | | | | | | | |
| --- | --- | --- | --- | --- | --- | --- | --- | --- |
| **Study characteristics** | | | **Population characteristics** | | | | | |
| **Author** | **Date** | **Design** | **Sample size** | **Gender (%)** | **Mean age (SD) (years)** | **Type of tinnitus** | **mean duration of tinnitus (SD) (years)** | **Presence hearing loss (%)** |
| ***Hyvärinen et al.*** | 2016 | RCT | IG 1: n=12 IG 2: n=15 IG 3: n=11 CG: n=5 | IG 1: ♂: 33% ♀: 67% IG 2: ♂: 67% ♀: 33% IG 3: ♂: 73% ♀: 27% CG: ♂: 20% ♀: 80% | IG 1: 56.3 (+/- NS) IG 2: 43.7 (+/-NS) IG 3: 51.9 (+/- NS) CG: 58.2 (+/- NS) | Chronic tinnitus (duration > 6 months) with TFI>18 | NS | IG 1: 41.7% IG 2: 26.7% IG 3: 36.4% CG: 80% |
| ***Schlee et al.*** | 2021 | SG  non-CT | n=39 | ♂: 45.5% ♀: 54.5% | 50.8(+/- 14.6) | Chronic tinnitus | 9.7 (+/-11.9) | NS |

RCT=Randomized Controlled Trial; non- CT= non-Controlled Trial; IG=Intervention Group; CG= Control Group; SD= Standard Deviation; NS= Not Specified; TFI= Tinnitus Functional Index

| ***Table S4: Study and population characteristics studies Self-help manuals*** | | | | | | | | |
| --- | --- | --- | --- | --- | --- | --- | --- | --- |
| **Study characteristics** | | | **Population characteristics** | | | | | |
| **Author** | **Date** | **Design** | **Sample size** | **Gender (%)** | **Mean age (SD) (years)** | **Type of tinnitus** | **mean duration of tinnitus (SD) (years)** | **Presence hearing loss (%)** |
| ***Kaldo et al.*** | 2007 | RCT | IG: n=34 CG: n=38 | IG: ♂: 50% ♀: 50% CG: ♂: 53% ♀: 47% | IG: 45.9 (+/- 13.0) CG: 48.5 (+/- 15.7) | Chronic tinnitus (duration > 6 months) with TRQ>10 | IG: 8.6 (+/- 8.4) CG: 12.4 (+/- 11.7) | IG: 71% CG: 65% |

RCT=Randomized Controlled Trial; IG=Intervention Group; CG= Control Group; SD= Standard Deviation; NS= Not Specified; TFI= Tinnitus Functional Index

| ***Table S5: Study and population characteristics studies Smartphone applications*** | | | | | | | | |
| --- | --- | --- | --- | --- | --- | --- | --- | --- |
| **Study characteristics** | | | **Population characteristics** | | | | | |
| **Author** | **Date** | **Design** | **Sample size** | **Gender (%)** | **Mean age (SD) (years)** | **Type of tinnitus** | **mean duration of tinnitus (SD) (years)** | **Presence hearing loss (%)** |
| ***Abouzari***  ***et al.*** | 2020 | RCT | IG: n=20 CG: n=10 | IG: ♂: 45% ♀: 55% CG: ♂: 70% ♀: 30% | IG: 54.2 (+/- 11.9) CG: 57.8 (+/- 10.9) | constant bilateral non-pulsatile chronic tinnitus (duration > 6 months) | NS | NS |
| ***Barozzi***  ***et al.*** | 2016 | RCT | IG1: n=19 IG2: n=17 | IG: ♂: 63.2% ♀: 36.8% CG: ♂: 70.6% ♀: 29.4% | IG: 54.77 (+/- 12.1) CG: 56.5 (+/- 11.6) | Chronic tinnitus (duration > 6 months) | NS | NS |
| ***Chatterjee***  ***et al.*** | 2021 | RCT | IG1: n=30 IG2: n=30 | IG1: ♂: 50% ♀: 50% IG2: ♂: 50% ♀: 50% | IG1:  ♂: 44.34(+/- 4.34)  ♀: 45.67 (+/- 3.38) IG2:  ♂:43.64 (+/- 4.34)  ♀: 46.43 (+/- 3.97) | NS | NS | Normal hearing sensitivity |
| ***Henry***  ***et al.*** | 2017 | SG  non-CT | n= 25 | ♂: 80% ♀: 20% | 53.1 (+/- 14.6) | NS | NS | NS |
| ***Kim***  ***et al.*** | 2016 | SG  non-CT | n=26 | ♂: 42.3% ♀: 57.7% | 51.4 (+/- 10.6) | Chronic tinnitus (duration > 3 months) with TFI>18 | 1.3 (+/-1.4) | NS |
| ***Kutyba***  ***et al.*** | 2021 | SG  non-CT | n=52 | ♂: 48% ♀: 52% | 48 (+/- 13.8) | Chronic Tinnitus | 5.4 (+/-5.9) | NS |
| ***Schlee***  ***et al.*** | 2021 | SG  non-CT | n=39 | ♂: 45.5% ♀: 54.5% | 50.8(+/- 14.6) | Chronic tinnitus | 9.7 (+/-11.9) | NS |
| ***Tyler***  ***et al.*** | 2018 | SG  non-CT | n=16 | ♂: 37.5% ♀: 62.5% | 56.75(+/- NS) | CI-patients with tinnitus (type not further specified) | NS | 100% (CI-patients) |

RCT=Randomized Controlled Trial; non- CT= non-Controlled Trial; IG=Intervention Group; CG= Control Group; SD= Standard Deviation; NS= Not Specified; TFI= Tinnitus Functional Index; CI= Cochlear Implant

| ***Table S6: Study and population characteristics studies Other internet-based interventions*** | | | | | | | | |
| --- | --- | --- | --- | --- | --- | --- | --- | --- |
| **Study characteristics** | | | **Population characteristics** | | | | | |
| **Author** | **Date** | **Design** | **Sample size** | **Gender (%)** | **Mean age (SD) (years)** | **Type of tinnitus** | **mean duration of tinnitus (SD) (years)** | **Presence hearing loss (%)** |
| ***Chattarjee et al.*** | 2021 | RCT | IG1: n=30 IG2: n=30 | IG1: ♂: 50% ♀: 50% IG2: ♂: 50% ♀: 50% | IG1: ♂: 44.34(+/- 4.34) ♀: 45.67 (+/- 3.38) IG2: ♂:43.64 (+/- 4.34) ♀: 46.43 (+/- 3.97) | NS | NS | NS |
| ***Hesser et al.*** | 2012 | RCT | IG 1: n=32 IG 2: n=35 CG: n=32 | IG 1: ♂: 56.2% ♀: 43.8% IG 2: ♂: 57.1% ♀: 42.9% CG: ♂: 56.2% ♀: 43.8% | IG 1: 48.8 (+/- 13.4) IG 2: 50.1 (+/-16.4) CG: 48.4(+/- 14.2) | Chronic tinnitus (duration > 6 months) with TFI>38 | NS | NS |

RCT=Randomized Controlled Trial; IG=Intervention Group; CG= Control Group; SD= Standard Deviation; NS= Not Specified; TFI= Tinnitus Functional Index
